# Expression of the SARS-CoV-2 Receptor ACE2 and Proinflammatory Cytokines Induced by the Periodontopathic Bacterium *Fusobacterium nucleatum* in Human Respiratory Epithelial Cells

**DOI:** 10.3390/ijms22031352

**Published:** 2021-01-29

**Authors:** Yuwa Takahashi, Norihisa Watanabe, Noriaki Kamio, Sho Yokoe, Ryuta Suzuki, Shuichi Sato, Toshimitsu Iinuma, Kenichi Imai

**Affiliations:** 1 Department of Complete Denture Prosthodontics, Nihon University School of Dentistry, Chiyoda-ku, Tokyo 101-8310, Japan; deyu18025@g.nihon-u.ac.jp (Y.T.); iinuma.toshimitsu@nihon-u.ac.jp (T.I.); 2 Department of Microbiology, Nihon University School of Dentistry, Chiyoda-ku, Tokyo 101-8310, Japan; watanabe.norihisa@nihon-u.ac.jp (N.W.); kamio.noriaki@nihon-u.ac.jp (N.K.); desh18040@g.nihon-u.ac.jp (S.Y.); dery18022@g.nihon-u.ac.jp (R.S.); 3 Department of Periodontology, Nihon University School of Dentistry, Chiyoda-ku, Tokyo 101-8310, Japan; satou.shuuichi@nihon-u.ac.jp; 4 Department of Oral Surgery, Nihon University School of Dentistry, Chiyoda-ku, Tokyo 101-8310, Japan

**Keywords:** ACE2, periodontitis, *Fusobacterium nucleatum*, proinflammatory cytokines, SARS-CoV-2, COVID-19

## Abstract

Coronavirus disease 2019 (COVID-19), caused by severe acute respiratory syndrome coronavirus 2 (SARS-CoV-2), is currently a global public health emergency. Periodontitis, the most prevalent disease that leads to tooth loss, is caused by infection by periodontopathic bacteria. Periodontitis is also a risk factor for pneumonia and the exacerbation of chronic obstructive pulmonary disease, presumably because of the aspiration of saliva contaminated with periodontopathic bacteria into the lower respiratory tract. Patients with these diseases have increased rates of COVID-19 aggravation and mortality. Because periodontopathic bacteria have been isolated from the bronchoalveolar lavage fluid of patients with COVID-19, periodontitis may be a risk factor for COVID-19 aggravation. However, the molecular links between periodontitis and COVID-19 have not been clarified. In this study, we found that the culture supernatant of the periodontopathic bacterium *Fusobacterium nucleatum* (CSF) upregulated the SARS-CoV-2 receptor angiotensin-converting enzyme 2 in A549 alveolar epithelial cells. In addition, CSF induced interleukin (IL)-6 and IL-8 production by both A549 and primary alveolar epithelial cells. CSF also strongly induced IL-6 and IL-8 expression by BEAS-2B bronchial epithelial cells and Detroit 562 pharyngeal epithelial cells. These results suggest that when patients with mild COVID-19 frequently aspirate periodontopathic bacteria, SARS-CoV-2 infection is promoted, and inflammation in the lower respiratory tract may become severe in the presence of viral pneumonia.

## 1. Introduction

Severe acute respiratory syndrome coronavirus 2 (SARS-CoV-2) is the cause of coronavirus disease 2019 (COVID-19) and is responsible for an ongoing pandemic. SARS-CoV-2 is a member of the human coronavirus family that targets the lower part of the respiratory tract. Its entry is induced through the binding of its viral spike protein to angiotensin-converting enzyme 2 (ACE2) as a host cellular receptor and is further triggered by host cell proteases, such as transmembrane protease serine 2 [1,2]. Recent studies suggested that in addition to direct lung injury by SARS-CoV-2, a hyper-inflammatory response, also referred to as the cytokine storm, contributes to disease severity in patients with COVID-19 [3,4,5]. In particular, increased levels of inflammatory markers, such as C-reactive protein and d-dimer, and increased levels of inflammatory cytokines, including interleukin (IL)-6, IL-8, and tumor necrosis factor (TNF)-α, are associated with excess inflammation, which increases the risk of mortality in patients with COVID-19 [3,4,5].

Periodontitis is one of the most pervasive diseases worldwide. It is a polymicrobial infection and multifactorial disease that is distinguished by chronic inflammation in the periodontium [6,7]. When untreated, this condition can cause alveolar bone destruction and may subsequently induce tooth loss, during which major periodontopathic bacteria, such as *Fusobacterium nucleatum, Porphyromonas gingivalis*, and *Prevotella intermedia*, participate in the induction of inflammation [6,7]. Recently, periodontopathic bacteria were revealed to be involved in the pathogenesis of respiratory diseases, such as aspiration pneumonia and chronic obstructive pulmonary disease (COPD), and other systemic diseases, including diabetes and cardiovascular disease [6,7]. Interestingly, such patients have increased rates of COVID-19 aggravation and mortality [8,9]. In addition, periodontopathic bacteria have been detected in the bronchoalveolar lavage fluid (BALF) of patients with pneumonia [10,11,12], and the risks of pneumonia and COPD are increased in patients with severe periodontal diseases [13,14,15]. Furthermore, we previously reported that periodontopathic bacteria can reactivate latent viruses, including human immunodeficiency virus (HIV)-1 and Epstein–Barr virus (EBV) [16,17,18]. In contrast, oral care, including periodontal treatment, can help prevent the onset and aggravation of aspiration pneumonia, COPD, and influenza [19,20,21]. In addition, periodontal treatment is effective for improving diabetes [22,23]. Interestingly, in a recent study, periodontopathic bacteria were detected in the BALF of patients with COVID-19 [24]. On the basis of the aforementioned observations, we hypothesized that increased counts of periodontopathic bacteria in the aspiration possibly induces ACE2 expression and proinflammatory cytokine production in the human respiratory epithelia.

In this study, we found that the culture supernatant of *F. nucleatum* (CSF) induced ACE2 expression in human alveolar epithelial cells and IL-6 and IL-8 production in human respiratory epithelial cells. These results suggest that increased counts of periodontopathic bacteria owing to poor oral hygiene contribute to the aggravation of COVID-19.

## 2. Results

### 2.1. CSF Stimulated ACE2 Expression in Human Alveolar Epithelial Cells

**Figure 1:**
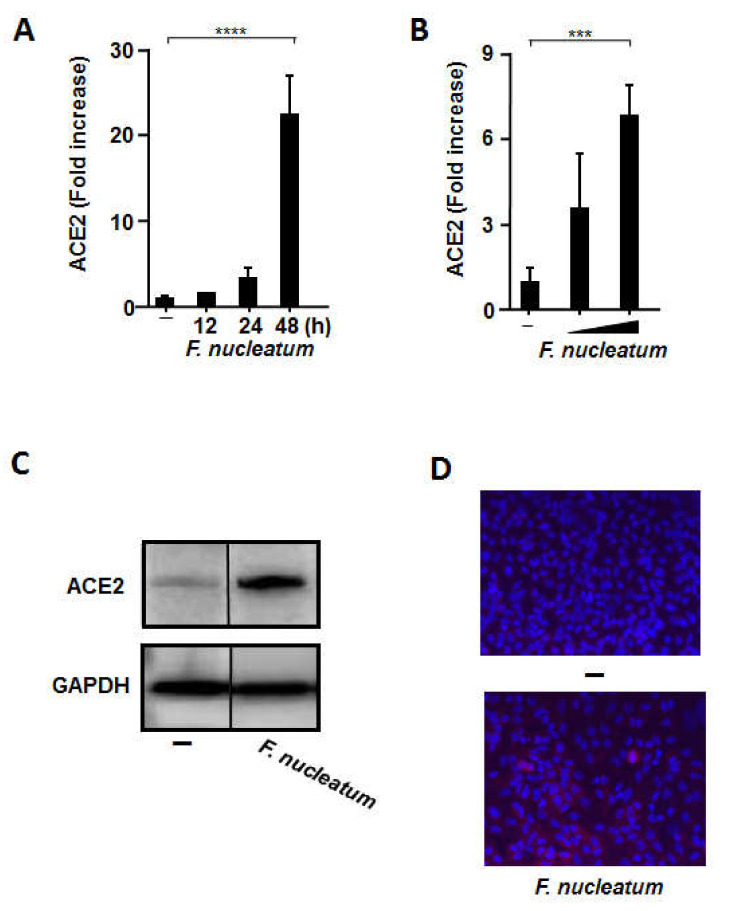
The culture supernatant of *Fusobacterium nucleatum* (CSF) upregulates angiotensin-converting enzyme 2 (ACE2) expression in A549 cells. A549 cells were incubated with or without CSF (100 µL/mL) for the indicated times (**A**) or at different concentrations (50 or 100 µL/mL) for 48 h (**B**). The cells were harvested, and *ACE2* mRNA levels were measured using RT-qPCR with specific primers. The mRNA levels were normalized to those of *glyceraldehyde-3-phosphate dehydrogenase* and expressed as fold increases. These experiments were conducted in triplicate, and data are presented as the mean ± SD. (**C**) A549 cells were treated with CSF (100 µL/mL) for 48 h. ACE2 protein expression was detected via Western blot analysis of whole cell lysates. (**D**) A549 cells were treated with CSF (100 μL/mL) for 24 h. ACE2 protein expression on A549 cells was detected via immunofluorescence analysis. Depicted are the merged images of ACE2 (red) and nucleus (blue). The values are presented as the mean ± SD; *n* = 3 (***, *p <* 0.005; ****, *p <* 0.0001).

To investigate whether CSF can upregulate *ACE2* transcription in A549 alveolar epithelial cells, we quantified the mRNA expression of *ACE2* using RT-qPCR. As presented in Figure 1A, a significant upregulation of *ACE2* mRNA expression was observed in the presence of CSF. *ACE2* mRNA levels increased after 24 h of exposure (3.3 ± 1.3-fold) to CSF and peaked after 48 h of incubation (22.4 ± 4.7-fold). *ACE2* expression in A549 cells was increased in a CSF concentration-dependent manner (Figure 1B). In addition, ACE2 protein expression was confirmed by Western blotting (Figure 1C) and immunofluorescence staining (Figure 1D) of A549 cells treated with CSF. ACE2 protein was distributed on the surface of A549 cells. In addition, we confirmed that brain heart infusion (BHI) broth does not affect the expression of ACE2 (Figure S1). Therefore, CSF could stimulate ACE2 mRNA and protein expression in A549 cells.

### 2.2. CSF Induced IL-8 and IL-6 mRNA Expression and Protein Production by Human Alveolar Epithelial Cells

**Figure 2:**
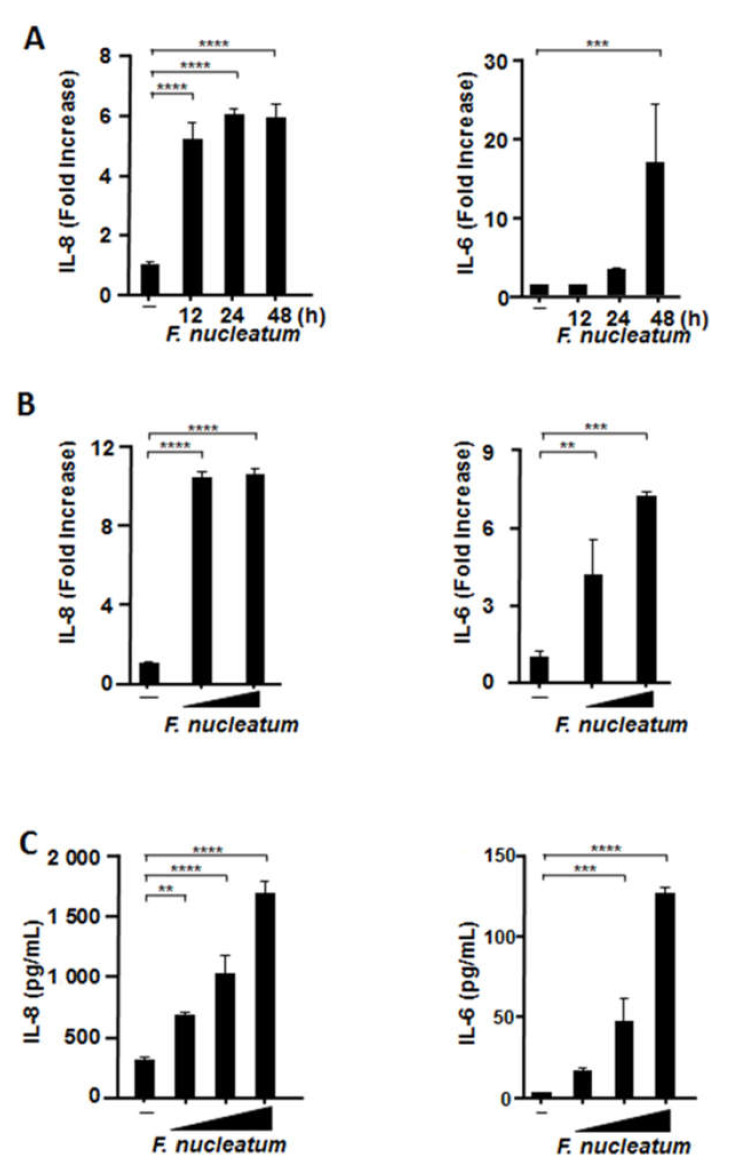
CSF induces the mRNA expression and protein production of proinflammatory cytokines in human alveolar epithelial cells. A549 cells were exposed to CSF (100 μL/mL) for the indicated times (**A**) or at different concentrations (50 or 100 µL/mL) for 48 h (**B**). The cells were harvested, and *interleukin* (*IL*)*-8* and *IL-6* mRNA levels were measured by RT-qPCR. The mRNA levels were normalized to those of glyceraldehyde-3-phosphate dehydrogenase and expressed as fold increases. (**C**) A549 cells were exposed to CSF (25, 50, or 100 µL/mL) for 48 h. IL-8 and IL-6 protein levels were ascertained by means of enzyme-linked immunosorbent assay and expressed as pg/mL. These experiments were performed in triplicate, and data are presented as the mean ± SD; *n* = 3 (**, *p <* 0.01; ***, *p <* 0.005; ****, *p <* 0.0001).

We then assessed the mRNA expression of *IL-8* and *IL-6* in A549 alveolar epithelial cells and pertinent protein release using RT-qPCR and enzyme-linked immunosorbent assay (ELISA), respectively. We confirmed that BHI does not affect the production of inflammatory cytokines in several respiratory epithelial cells (Figure S1). As illustrated in Figure 2A, CSF upregulated *IL-8* mRNA expression after 12 h of exposure (5.2 ± 0.6-fold). CSF also upregulated *IL-6* mRNA expression after 24 h, with the expression peaking after 48 h (16.9 ± 7.7-fold). In addition, Figure 2B,C demonstrates that CSF elicited the mRNA expression and production of both cytokines in a CSF concentration-dependent manner.

### 2.3. CSF Induced IL-8 and IL-6 Production by Human Primary Alveolar Epithelial Cells

**Figure 3:**
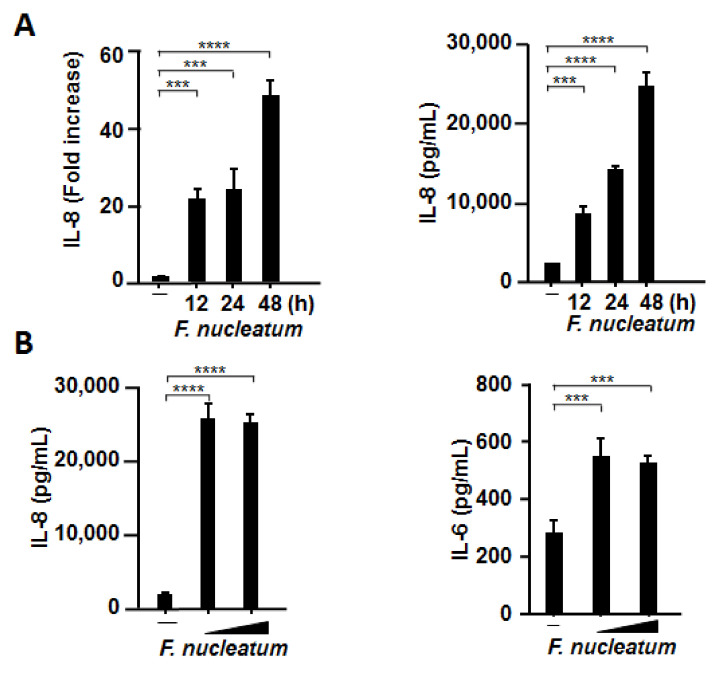
CSF induces mRNA expression and protein production of proinflammatory cytokines by primary human alveolar epithelial cells. Primary human alveolar epithelial cells were exposed to CSF (100 μL/mL) for the indicated times (**A**) or at different concentrations (50 or 100 µL/mL) for 48 h (**B**). The cells were harvested, and *IL-8* and *IL-6* mRNA levels were assessed using RT-qPCR. The mRNA levels were normalized to those of *glyceraldehyde-3-phosphate dehydrogenase* and expressed as fold increases. IL-8 and IL-6 protein levels were determined by means of enzyme-linked immunosorbent assay and expressed as pg/mL. These experiments were conducted in triplicate, and data are presented as the mean ± SD; *n* = 3 (***, *p <* 0.005; ****, *p <* 0.0001).

Since it was recommended to utilize primary cells because of their numerous advantages, we confirmed whether CSF induces the production of IL-8 and IL-6 in primary alveolar epithelial cells. As highlighted in Figure 3A, CSF strongly induced the mRNA expression and production of IL-8 in a time-dependent manner. In addition, CSF induced IL-8 and IL-6 production more strongly in primary cells than in A549 cells at the same concentration (Figure 3B).

### 2.4. CSF Induced IL-8 and IL-6 Production by Human Bronchial Epithelial Cells and Human Pharyngeal Epithelial Cells

**Figure 4:**
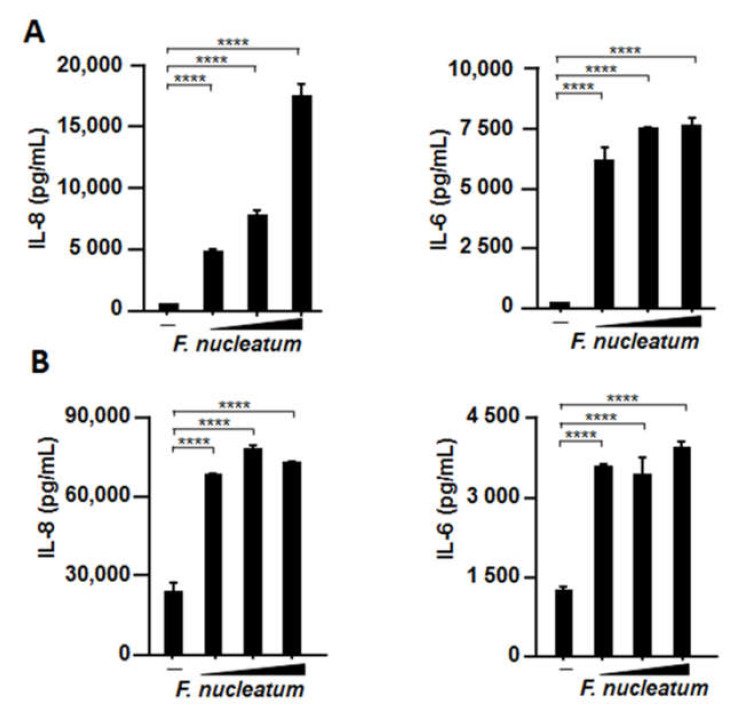
Effects of CSF on IL-8 and IL-6 production by two human respiratory epithelial cells. Human bronchial (BEAS-2B) (**A**) and pharyngeal (Detroit 562) (**B**) epithelial cells were incubated with CSF (25, 50, or 100 µL/mL) for 48 h. IL-8 and IL-6 protein levels in the cell culture supernatants were determined by means of enzyme-linked immunosorbent assay. These experiments were conducted in triplicate, and data are presented as the mean ± SD; *n* = 3 (****, *p <* 0.0001).

Because CSF induced IL-8 and IL-6 production in A549 cells and primary cells, we examined whether other human respiratory epithelial cells, such as BEAS-2B human bronchial epithelial cells and Detroit 562 human pharyngeal cells, also responded to the stimulation by periodontopathic bacteria and their products because all swallowed materials are retained in the pharynx for some time. As was expected, higher IL-8 and IL-6 production was induced by CSF in both BEAS-2B (Figure 4A) and Detroit 562 cells (Figure 4B).

### 2.5. Effects of Butyric Acid (BA) on the Expression of ACE2 and Proinflammatory Cytokines in Human Respiratory Epithelial Cells

**Figure 5:**
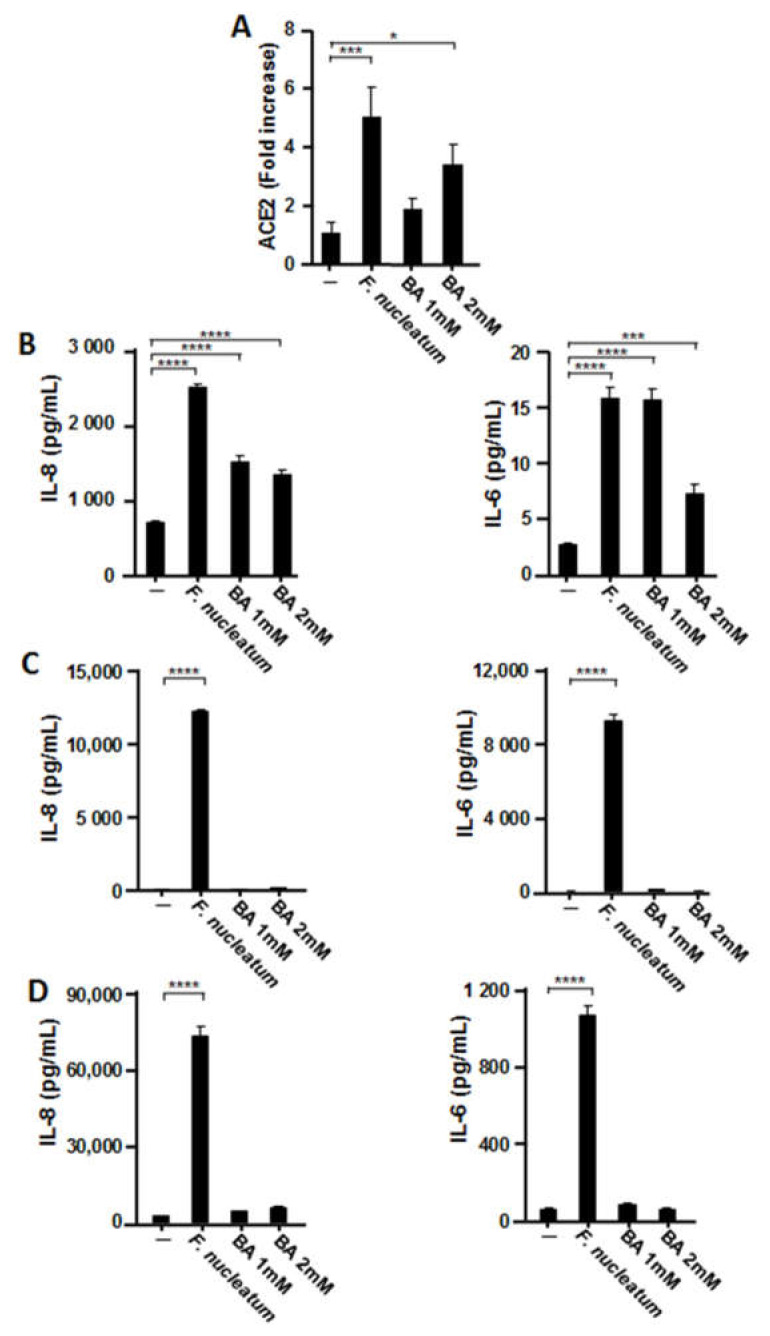
Effects of butyric acid (BA) on ACE2 and proinflammatory cytokine expression in respiratory epithelial cells. A549 cells were incubated with CSF (100 µL/mL) or BA (1 or 2 mM) for 48 h (**A**). The mRNA levels were normalized to those of *glyceraldehyde-3-phosphate dehydrogenase* and expressed as fold increases. A549 (**B**), BEAS-2B (**C**), and Detroit 562 (**D**) epithelial cells were incubated with CSF (100 µL/mL) or BA (1 or 2 mM) for 48 h. IL-8 and IL-6 protein levels in the cell culture supernatants were determined via enzyme-linked immunosorbent assay. These experiments were conducted in triplicate, and data are presented as the mean ± SD; *n* = 3 (*, *p <* 0.05; ***, *p <* 0.005; ****, *p <* 0.0001).

As an important virulence factor, BA is produced by *F. nucleatum* in large quantities during anaerobic glycolysis. Our studies indicated that BA plays a critical role in a variety of diseases, including viral infections such as those caused by HIV-1 and EBV [16,17,18]. BA inhibits the enzymatic activity of histone deacetylase (HDAC) by competing with HDAC substrates for the enzyme’s active site pocket, which contains the catalytic center [25], thereby stimulating the transcription of various genes [26,27]. Therefore, we examined the effects of BA on *ACE2* expression and inflammatory cytokine production. As presented in Figure 5A, BA induced *ACE2* expression, but the extent of the expression was approximately half that induced by CSF. Next, we investigated whether BA could induce IL-8 and IL-6 expression by respiratory epithelial cells. As illustrated in Figure 5B, the production of both proinflammatory cytokines was observed in BA-treated A549 cells. Conversely, proinflammatory cytokine production was not induced by CSF in BEAS-2B and Detroit 562 cells (Figure 5C,D).

## 3. Discussion

Periodontitis is a risk factor for pneumonia and the exacerbation of COPD, presumably because of the aspiration of saliva contaminated with periodontopathic bacteria and their products into the lower respiratory tract [11,13,15,28,29]. In addition, approximately half of healthy adults experience salivary aspiration during sleep, but the risk of aspiration is particularly increased in elderly people because of reduced laryngopharyngeal sensitivity, which results in impaired cough and swallowing reflexes [30,31,32]. This may allow saliva contaminated with periodontopathic bacteria and their products to be more continually aspirated into the lower respiratory tract. It is more likely for COVID-19 to be severe in elderly people and medically compromised patients [8,9], who have a higher risk of aspiration because of a decreased swallowing function. Indeed, periodontopathic bacteria, which are not indigenous constituents of the lower respiratory microflora, have been isolated from the BALF of patients with COVID-19 [24]. Therefore, periodontitis may be considered a risk factor for COVID-19 aggravation. However, the molecular links between periodontitis and COVID-19 have not been clarified. To the best of our knowledge, we have presented for the first time that ACE2 expression was induced by other microorganisms at the cellular level.

It is important for a virus or bacterium to bind to a host cellular receptor to cause infection. ACE2 expression is enhanced by stimuli such as smoking [33]. Ziegler et al. reported that ACE2 expression was elevated in people infected with HIV and influenza virus [34]. Together with these reports, our results suggest that when periodontopathic bacteria are aspirated, ACE2 expression is increased in the lungs because of stimulation by periodontopathic bacteria and their pathogenic factors. Increased ACE2 expression in the lungs, as promoted by periodontopathic bacteria, may increase SARS-CoV-2 infection rate in the lungs. In fact, periodontopathic bacteria enhance the expression of influenza virus receptor [35] and platelet-activating factor receptor, which are the receptors for etiological bacteria of pneumonia such as *Streptococcus pneumoniae* and *Pseudomonas aeruginosa* [36]. In addition, the concept that aspiration of another periodontopathic bacterium, namely, *P. gingivalis*, into the lungs and periodontal disease-associated enzymes in the saliva may modify mucosal surfaces to promote adhesion and colonization by respiratory pathogens such as *Haemophilus influenzae* has been postulated [37]. Although we confirmed that CSF also induces ACE2 expression in the lungs of mice (data not shown), it is important to assess whether the induction of ACE2 by CSF actually promotes SARS-CoV-2 infection.

Several studies revealed that the cytokine storm induced by severe SARS-CoV-2 is a major cause of disease severity and death [3,4,5]. In particular, IL-8, IL-6, and TNF-α levels were found to be significantly elevated in the sera of patients with COVID-19 compared with those of healthy donors or with plasma isolated from chimeric antigen receptor-modified T cell-treated patients with no cytokine release syndrome [4]. We found that CSF induced IL-8 and IL-6 production by A549 alveolar epithelial cells and primary alveolar epithelial cells. In addition, CSF also strongly induced IL-8 and IL-6 by BEAS-2B bronchial epithelial cells and Detroit 562 pharyngeal epithelial cells. Although CSF induced *TNF-α* mRNA expression, the induction at the protein level was extremely weak in all epithelial cell lines (data not shown). IL-8 is a potent proinflammatory cytokine playing a key role in the recruitment and activation of neutrophils during inflammation [38,39], and given the frequency of neutrophilia observed in patients infected with SARS-CoV-2, it is possible that IL-8 contributes to the pathophysiology of COVID-19.

In many chronic inflammatory diseases, IL-6 is involved in the stimulation of acute-phase protein synthesis, leukocyte recruitment, B cell differentiation, and T cell activation [39]. Trials to block IL-6 signaling with drugs that have already been approved by the FDA have been launched worldwide, and some clinical benefits have been observed in a subset of patients in small, single-center, observational studies [40]. As IL-8 and IL-6 exert their proinflammatory effects in paracrine and autocrine manners, it is crucial that they elicit inflammatory responses in the neighboring host cells. Our findings thus indicate that inflammation in the lower respiratory epithelial cells is caused not only by exposure to CSF itself but also by the absorption of paracrine proinflammatory cytokines released from the pharyngeal epithelium, which is induced by precedent exposure to CSF. We also reported that heat-inactivated periodontopathic bacteria can induce the production of proinflammatory cytokines in respiratory epithelial cells also *in vivo*, in which these bacteria more strongly induce cytokine production than *S. pneumoniae* [41,42,43]. Therefore, periodontopathic bacteria are potent proinflammatory stimulants of the lower respiratory tract through aspiration.

SARS and Middle East respiratory syndrome symptoms may be aggravated by combined infection by viruses and bacteria [44,45]. Similarly, secondary bacterial infection has also been suggested to be involved in the aggravation of COVID-19 [46,47]. Because periodontopathic bacteria have been observed in the BALF of patients with severe COVID-19 [24], the frequent aspiration of periodontopathic bacteria in patients with mild COVID-19 might exacerbate COVID-19 symptoms together with viral pneumonia because of the induction of ACE2 and proinflammatory cytokines by the bacteria.

*F. nucleatum* is a common anaerobic bacterium responsible for periodontitis and has also been identified as an etiologic pathogen of gastrointestinal and respiratory anaerobic infection. Although we used CSF as a stimulant in this study, it is unclear what substances in CSF induced ACE2 and proinflammatory cytokine expression. However, our data suggest that BA in CSF may be partially responsible for some of the observed effects in A549 alveolar epithelial cells.

We previously demonstrated that CSF contains BA in high concentrations (6.7–27 mM) and that it can inhibit HDACs, thereby increasing the level of histone acetylation and the transcriptional activity of the HIV-1 and EBV lytic switch transactivator BZLF1 [16,17,18]. It has been reported that more than adequate concentrations of BA for gene activation were present in the dental plaques (range = 4.7–13.8 mM) and periodontal pockets (mean, 2.6 ± 0.4 mM) of patients with periodontal disease [48,49], whereas BA concentration was below the detection limits in healthy sites [50]. We also previously reported that high concentrations (millimolar levels) of BA are present in the saliva of Japanese patients with periodontal disease, and a significant correlation between BA concentrations and *BZLF1* transcript levels was noted [51]. These observations and our findings suggest that BA produced by *F. nucleatum* in the saliva of patients with periodontal disease might be involved in the upregulation of ACE2 and proinflammatory cytokines. However, because proinflammatory cytokine production was not induced by BA in BEAS-2B and Detroit 562 cells, BA receptor expression levels and the ability of BA to influence intracellular signals may differ by cell type. In addition to BA, various factors of *F. nucleatum* such as *Fusobacterium nucleatum* adhesin A (FadA) and lipopolysaccharides (LPS) may affect gene expression. Furthermore, although only CSF was used in this study, we previously reported that another periodontopathic bacterium, *P. gingivalis*, also produces high concentrations of BA [16,17,18]. Therefore, further studies are needed to clarify the mechanism by which the CSF and BA of periodontopathic bacteria induce ACE2 and proinflammatory cytokine expression.

The current focus of COVID-19 research has been on the development of novel therapeutics, including antivirals and vaccines. However, effective vaccines and drugs have not yet been developed. The reduced likelihood of professional oral care associated with the long-term hospitalization of patients with COVID-19 may increase the risk of inflammation in the lower respiratory tract. Although further studies are needed, our results provide the novel finding that increased prevalence of periodontopathic bacteria associated with poor oral hygiene may contribute to the aggravation of COVID-19 via ACE2 and proinflammatory cytokine secretion [52]. Therefore, good oral hygiene for reducing the amount of aspirated oral bacteria can potentially prevent COVID-19 exacerbation.

## 4. Materials and Methods

### 4.1. Cell Culture

Human alveolar (A549), bronchial (BEAS-2B), and pharyngeal (Detroit 562) epithelial cells were purchased from ATCC (Manassas, VA, USA) and maintained at 37 °C in Dulbecco’s modified Eagle’s medium (Sigma-Aldrich, St. Louis, MO, USA) consisting of 10% heat-inactivated fetal bovine serum (Thermo Fisher Scientific, Rockford, IL, USA), penicillin (100 U/mL), and streptomycin (100 μg/mL), as described previously [41,42].

### 4.2. Primary Human Alveolar Epithelial Cell Culture

Primary human alveolar epithelial cells (ScienCell Research Laboratories, Carlsbad, CA, USA) were cultured in alveolar epithelial cell medium with growth supplement following the manufacturer’s recommendations. Primary cells, growth medium, and growth supplement were purchased from ScienCell Research Laboratories. Briefly, primary cells were grown at 37 °C for 48 h in a CO_2_ chamber. Cell growth and concentration were visually confirmed using a microscope and through optical density.

### 4.3. Bacterial Strains and Culture

The *F. nucleatum* strain ATCC 25586 was cultured in BHI broth (Becton, Dickinson and Company, Sparks, MD, USA) that was supplemented with 5 μg/mL hemin and 0.5 μg/mL menadione. Bacterial cultures were grown in an anaerobic system (5% CO_2_, 10% H_2_, and 85% N_2_ at 37 °C using a model 1024 anaerobic chamber; Forma Scientific) for 48 h [41]. Subsequently, CSF was collected via centrifugation at 10,000× *g* at 4 °C for 20 min and filter-sterilized through a 0.22 µm pore size membrane filter to remove bacterial cells. CSF pH was likewise determined and found to be within 6.8–7.0.

### 4.4. mRNA Preparation and RT-qPCR Assay

Cells were stimulated with CSF (2.5 (25 µL/mL), 5 (50 µL/mL), or 10% (100 µL/mL) *v/v*) or BA (1 or 2 mM) and incubated at 37 °C. The experimental procedures for RNA purification and RT-qPCR assay were performed as previously described [43]. Briefly, cells were washed once with ice-cold phosphate-buffered saline (PBS) and homogenized using a QIAshredder (Qiagen, Hilden, Germany), whereas total RNA was purified using an RNeasy Mini Kit (Qiagen) according to the manufacturer’s instructions. For cDNA synthesis, total RNA was reverse-transcribed using an RNA PCR kit (PrimeScript; Takara Bio, Shiga, Japan). The resulting cDNA mixture was subjected to real-time PCR using SYBR Premix Ex Taq solution (Takara Bio) containing 0.2 μM sense and antisense primers. The primer sequences used for the amplification of each gene were as follows: *ACE2*, forward (5′-CAT TGG AGC AAG TGT TGG ATC TT-3′) and reverse (5′-GAG CTA ATG CAT GCC ATT CTC A-3′); *IL-8*, forward (5′-CTT GTC ATT GCC AGC TGT GT-3′) and reverse (5′-TGA CTG TGG AGT TTT GGC TG-3′); *IL-6*, forward (5′-TTC GGT CCA GTT GCC TTC TC-3′) and reverse (5′-GAG GTG AGT GGC TGT CTG TG-3′); and *glyceraldehyde-3-phosphate dehydrogenase* (*GAPDH*), forward (5′-ACC AGC CCC AGC AAG AGC ACA AG-3′) and reverse (5′-TTC AAG GGG TCT ACA TGG CAA CTG-3′). PCR assays were performed using a TP-800 Thermal Cycler Dice Real-Time System (Takara Bio). All real-time PCR experiments were performed in triplicate, and the specificity of each product was verified by means of melting curve analysis. The calculated gene expression levels were normalized to *GAPDH* mRNA levels.

### 4.5. Western Blot Analysis

The experimental procedures for Western blotting were described previously [16]. Briefly, after treatment with CSF for 48 h, the cells were washed with PBS and lysed using RIPA buffer. Equal amounts of protein (20 μg) were resolved on a 10% sodium dodecyl sulfate-polyacrylamide gel and transferred onto a polyvinylidene fluoride membrane (EMD Millipore Corporation, Billerica, MA, USA). The membranes were blocked with 5% skim milk and incubated with primary antibodies overnight at 4 °C. The primary antibodies included anti-ACE2 (Abcam, Cambridge, MA, USA) and anti- GAPDH antibodies (Santa Cruz Biotechnology, Santa Cruz, CA, USA). The membranes were washed with Tris-buffered saline containing Tween 20 and incubated with a secondary antibody for 1 h at room temperature. After washing, protein bands were detected using the ChemiDoc XRS System (Bio-Rad, Hercules, CA, USA).

### 4.6. Immunofluorescence

A549 cells grown on cover slides pretreated with poly-l-lysine (Matsunami Glass, Osaka, Japan) were treated with CSF for 48 h. The slides were washed with PBS and then fixed with 4% paraformaldehyde (Wako Pure Chemical Industries, Osaka, Japan) for 15 min at room temperature. The slides were blocked with 2% bovine serum albumin (BSA) for 1 h and incubated with anti-ACE2 antibody (R&D Systems, Minneapolis, MN, USA) in blocking buffer overnight at 4 °C. After thorough rinsing with PBS, the slides were incubated with Alexa Fluor 555 anti-goat secondary antibody (Abcam, Cambridge, MA, USA) in PBS containing 2% BSA and Hoechst 33342 (Thermo Fisher Scientific). The cover slides were mounted on glass slides using the ProLong Gold antifade reagent (Thermo Fisher Scientific) and examined using a fluorescence microscope (IX-FLA, Olympus, Tokyo, Japan). The images obtained were subsequently analyzed using InStudio (Pixera, Santa Clara, CA, USA).

### 4.7. IL-8 and IL-6 Measurements

IL-8 and IL-6 concentrations in the cell culture supernatants were measured using an ELISA kit (R&D Systems) according to the procedures recommended by the manufacturer. All experiments were performed in triplicate, and data are presented as the mean ± standard deviation.

### 4.8. Statistical Analysis

Data are presented as the mean ± SD. Statistical analysis was performed using one-way analysis of variance with Tukey’s multiple comparisons test; *p* < 0.05 was considered statistically significant.

## Data Availability

The data presented in this study are available in the article.

## Supplementary Materials

The following are available online at https://www.mdpi.com/1422-0067/22/3/1352/s1, Figure S1: Effect of BHI on the expression of ACE2 and the production of inflammatory cytokines.
