# Supplementary material for: Expression of the SARS-CoV-2 Receptor ACE2 and Proinflammatory Cytokines Induced by the Periodontopathic Bacterium Fusobacterium nucleatum in Human Respiratory Epithelial Cells
Source: Int J Mol Sci. 2021 Jan 29;22(3):1352. doi: 10.3390/ijms22031352 (PMC7866373; doi:10.3390/ijms22031352)
Supplement: Supplementary file 1 [file ijms-22-01352-s001.pdf]

Supplemental Material

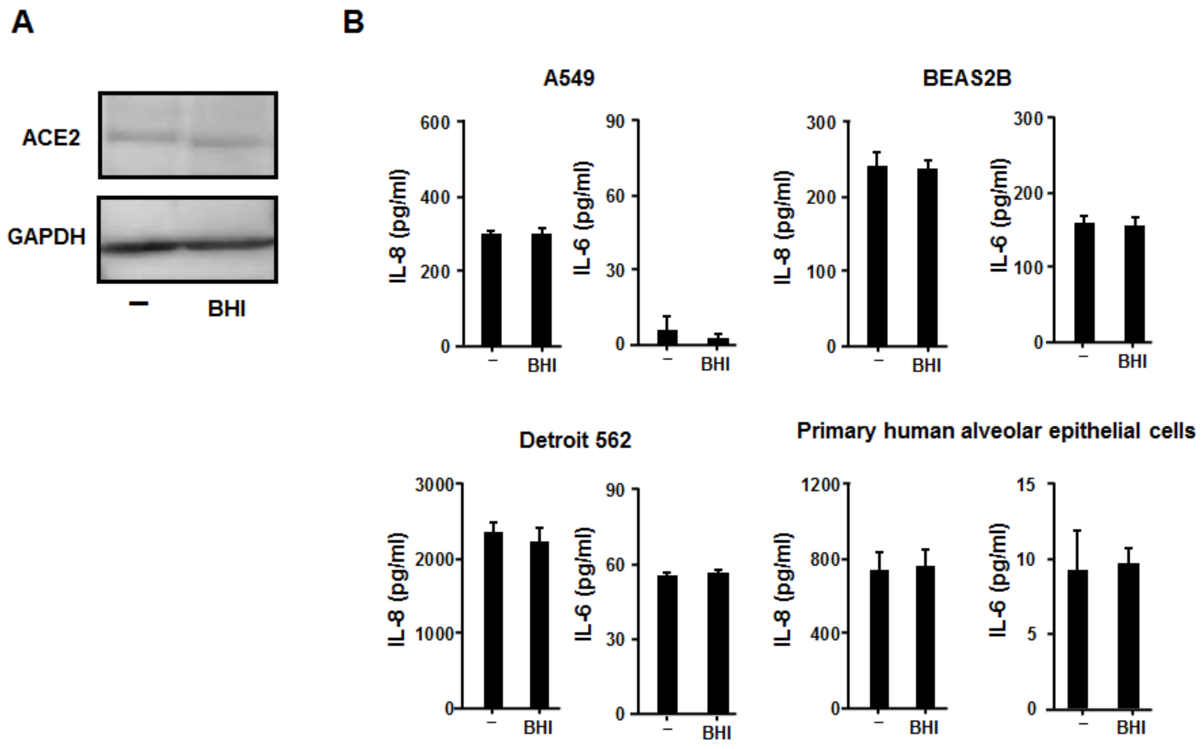

Figure S1. Effect of brain heart infusion (BHI) on expression of ACE2 and production of inflammatory cytokines. (**a**) A549 cells were treated with BHI (100 µl/ml) for 48 h. ACE2 protein expression was detected via Western blot analysis of whole cell lysates. (**b**) Several respiratory epithelial cells were incubated with BHI (100 µl/ml) for 48 h. IL-8 and IL-6 protein levels in the cell culture supernatants were determined by means of enzyme-linked immunosorbent assay. These experiments were conducted in triplicate, and data have been presented as the mean ± SD; *n* = 3.
